# First-in-Human Abdominal Aortic Aneurysms Trial with Tricaprin (F-HAAAT): Study Design and Protocol

**DOI:** 10.1016/j.cjco.2024.10.010

**Published:** 2024-11-05

**Authors:** Takahito Kamba, Masahiro Yanagawa, Kazuo Shimamura, Satoshi Yamaguchi, Kenji Shirakura, Satomi Okamura, Yuki Nishimura, Tomomi Yamada, Yasushi Sakata, Noriyuki Tomiyama, Shigeru Miyagawa, Ken-ichi Hirano, Nobuhiro Zaima

**Affiliations:** a Laboratory of Cardiovascular Disease, Novel, Non-Invasive, and Nutritional Therapeutics (CNT), Department of Triglyceride Science, Graduate School of Medicine, Osaka University, Osaka, Japan; b Department of Cardiovascular Medicine, Osaka University Graduate School of Medicine, Osaka, Japan; c Department of Radiology, Osaka University Graduate School of Medicine, Osaka, Japan; d Department of Cardiovascular Surgery, Osaka University Graduate School of Medicine, Osaka, Japan; e Tochino Foundation, Osaka, Japan; f Medical Center for Translational Research, Department of Medical Innovation, Osaka University Hospital, Osaka, Japan; g Data Coordinating Center, Department of Medical Innovation, Osaka University Hospital, Osaka, Japan; h The Laboratory of Applied Cell Biology, Faculty of Agriculture, Kindai University, Nara, Japan; i Agricultural Technology and Innovation Research Institute, Kindai University, Nara, Japan

## Abstract

Approximately 2%-12% of individuals aged > 65 years worldwide are estimated to have an abdominal aortic aneurysm (AAA), with a mortality rate exceeding 60% in rupture cases. The sole preventive intervention against rupture is timely surgery, which requires substantial medical resources, including postoperative complication management. Although numerous randomized clinical trials have been performed, no oral medication effectively treats AAA. Tricaprin, a medium-chain triglyceride with 3 capric acids, is used in dietary therapy for metabolic and neurological disorders. Our group recently reported that tricaprin, unlike other medium-chain triglycerides, showed reverse remodelliing of AAA in a rat model. Determining whether this basic finding could be translated to clinical practice is important. The **F**irst-in-**H**uman **A**bdominal **A**ortic **A**neurysms trial with **T**ricaprin (F-HAAAT) proposes the first-in-human AAA trial to confirm the safety of tricaprin use in patients with small AAA, exploring novel assessment methods to evaluate treatment efficacy. This single-centre, open-label, single-arm study will include 10 patients (aged 50–85 years) with small AAA (30–45 mm in diameter) receiving daily oral tricaprin (1.5–3.0 g/d) for 52 weeks. Primary endpoints include safety evaluation of tricaprin determined by monitoring all adverse events, particularly major adverse cardiovascular events, AAA-related adverse events, and other unpredictable events. Secondary endpoints include parameters to validate tricaprin efficacy by measuring AAA diameter, volume, and Agatston score, and analyzing computed tomography values of the aortic aneurysmal wall. Outcomes of the trial may provide insights into noninvasive methods for indirectly analyzing AAA pathologic characteristics and revealing aneurysmal reverse remodelliing (jRCTs051240036, Japan Registry of Clinical Trials).

## Graphical abstract

**Figure:**
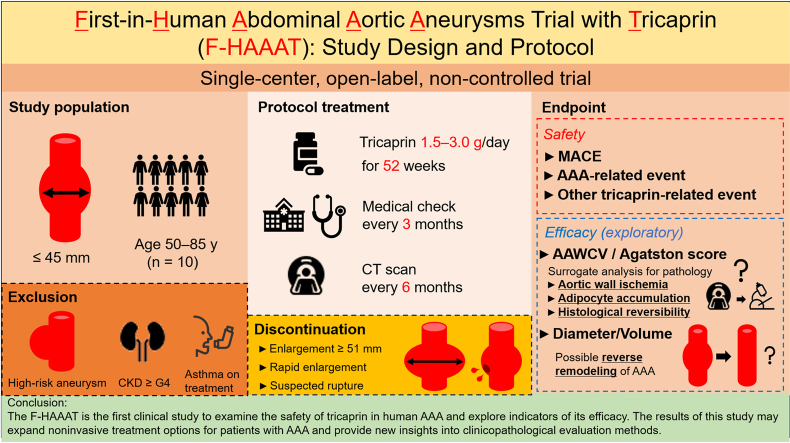

## Résumé

On estime qu'environ 2 à 12 % des personnes âgées de plus de 65 ans dans le monde sont atteintes d'un anévrisme de l'aorte abdominale (AAA), avec un taux de mortalité supérieur à 60 % en cas de rupture. La seule intervention préventive contre cette rupture est une intervention chirurgicale opportune, qui nécessite des ressources médicales considérables, y compris la gestion des complications postopératoires. Bien que de nombreux essais cliniques randomisés aient été réalisés, aucun médicament oral ne traite efficacement l'AAA. La tricaprine, un triglycéride à chaîne moyenne contenant 3 acides capriques, est utilisée dans la thérapie diététique pour les troubles métaboliques et neurologiques. Notre groupe a récemment rapporté que la tricaprine, contrairement à d'autres triglycérides à chaîne moyenne, présentait un remodelage inversé de l'AAA dans un modèle de rat. Il est important de déterminer si cette découverte fondamentale peut être transposée à la pratique clinique. L'essai clinique F-HAAAT (pour **F**irst-in**-H**uman **A**bdominal **A**ortic **A**neurysms trial with **T**ricaprin) propose le premier essai sur l'AAA chez l'Homme pour confirmer la sécurité de l'utilisation de la tricaprine chez les patients souffrant d'un petit AAA, en explorant de nouvelles méthodes d'évaluation pour attester de l'efficacité du traitement. Cette étude monocentrique, ouverte et à un seul bras, inclura 10 patients (âgés de 50 à 85 ans) souffrant d'un petit AAA (30 à 45 mm de diamètre) recevant quotidiennement de la tricaprine par voie orale (1,5 à 3,0 g/j) pendant 52 semaines. Les principaux critères d'évaluation incluent l'évaluation de la sécurité du tricaprine déterminée en surveillant tous les événements indésirables, en particulier les événements cardiovasculaires indésirables majeurs, les événements indésirables liés à l'AAA et d'autres événements imprévisibles. Les critères d'évaluation secondaires comprennent des paramètres permettant de valider l'efficacité de la tricaprine en mesurant le diamètre de l'AAA, son volume et le score calcique d'Agatston, et en analysant les valeurs de la paroi anévrismale de l'aorte obtenues par tomographie assistée par ordinateur. Les résultats de l'essai peuvent fournir des indications sur les méthodes non invasives permettant d'analyser indirectement les caractéristiques pathologiques de l'AAA et de révéler le remodelage inverse de l'anévrisme (jRCTs051240036, Registre japonais des essais cliniques).

Abdominal aortic aneurysm (AAA) is a prevalent cardiovascular condition affecting approximately 2%-12% of individuals aged > 65 years worldwide.^1^ Left untreated, AAA can progressively expand asymptomatically, culminating in a potentially fatal rupture in 0.04 persons/100 person-years^2^ or < 5%^3^ per year in those with small AAA.^4^ Current treatment options primarily involve surgical interventions, such as open surgical repair or endovascular aneurysm repair, which carry inherent risks and are associated with long-term complications.^5^ Recent data indicate a 30-day mortality rate of 24.7% after emergency surgery for ruptured AAA, with a major adverse cardiovascular events (MACE) rate of 20.2%. In contrast, the 30-day mortality rate for elective surgery in unruptured AAA ranges from 1.0% in patients aged < 70 years to 10%, even in those aged > 70 years, underscoring the importance of preventing AAA rupture through use of medication.^6^, ^7^, ^8^ Thus, a considerable unmet medical need is for effective and noninvasive therapies for AAA management.

Developing noninvasive AAA treatments requires identifying the mechanisms and active compounds that bridge the translational gap between animal studies and clinical practice. Despite advancements in the understanding and imaging of AAA pathophysiology, previous randomized controlled trials^9^, ^10^, ^11^, ^12^, ^13^, ^14^, ^15^, ^16^ in humans have failed to translate the therapeutic effects observed in conventional animal models—such as those using angiotensin II injection, porcine pancreatic elastase administration, and calcium salts application^17^—into clinical success. In the course of resolving this translational gap, through the findings obtained from the analyses of human AAA specimens, we found that inducing hypoperfusion in the abdominal aortic wall can induce AAA in rats.^18^ This model closely resembles human AAA, with vasa vasorum stenosis, increased vasa vasorum, ectopic adipocytes, intraluminal thrombi, and a high rupture rate. Aortic hypoperfusion with ischemic reaction, also described in human AAA, is now recognized as a critical pathogenic mechanism^19^ that could contribute significantly to AAA formation. We previously validated the influence of several agents^20^, ^21^, ^22^, ^23^, ^24^, ^25^ on aortic degeneration in the context of seeking medical treatment for AAA. Among these candidates, our 2023 report on the effect of tricaprin on preventing AAA development presented notable results. In an animal aortic hypoperfusion model, oral administration of tricaprin before the AAA induction procedure almost completely abrogated the induction of AAA without resolving the hypoperfusion of the aortic wall.^26^ Tricaprin, composed of 3 capric acids (C-10 medium-chain fatty acids) and 1 glycerol, is a nutritional ingredient in breast milk^27^ and coconut oil.^28^ We reported that tricaprin had a promising effect on myocardial lipolysis^29^ and diffuse coronary stenosis^30^ in patients with triglyceride deposit cardiomyovasculopathy. Tricaprin reduces excessive triglyceride accumulation in vascular smooth muscle cells. Given that triglyceride accumulation in the adventitia of AAA has been confirmed and is associated with rupture rate,^31^ tricaprin use may be effective in treating vascular diseases, including AAA.

Building on the pathologic features of human AAA, findings from the aortic hypoperfusion rodent model, and results from the tricaprin experiment, we designed the First-in-Human Abdominal Aortic Aneurysms trial with Tricaprin (F-HAAAT). This study aims to evaluate the safety of tricaprin use in patients with AAA and explore potential indicators of its efficacy. By analyzing changes in the Agatston score and aortic aneurysm wall, computed tomography values (AAWCVs) before and after tricaprin administration, we aim to characterize the histologic changes within the AAA wall comprehensively. This goal includes evaluating classical parameters, such as aneurysm diameter and volume, focusing on the presence of aortic perfusion defects. The AAWCV method was developed to noninvasively analyze the pathology of AAA by classifying the computed tomography (CT) values of AAA components into 6 groups and monitoring the volume changes in each category to indirectly assess histologic changes. Although the reverse remodelliing effect of tricaprin on AAA remains uncertain, owing to the small number of participants and short evaluation duration in this trial, we expect that AAWCV will be a suitable indicator for evaluating histologic alterations. The F-HAAAT study was designed to establish a new paradigm for AAA treatment, based on scientific evaluation and innovative imaging techniques.

## Materials and Methods

### Materials

CAPRINHI, a dietary supplement containing 99.2% tricaprin, with one tablet (400 mg) being equivalent to 250 mg of tricaprin, was provided by Tochino Foundation (Osaka, Japan).

### Study design

**Figure 1:**
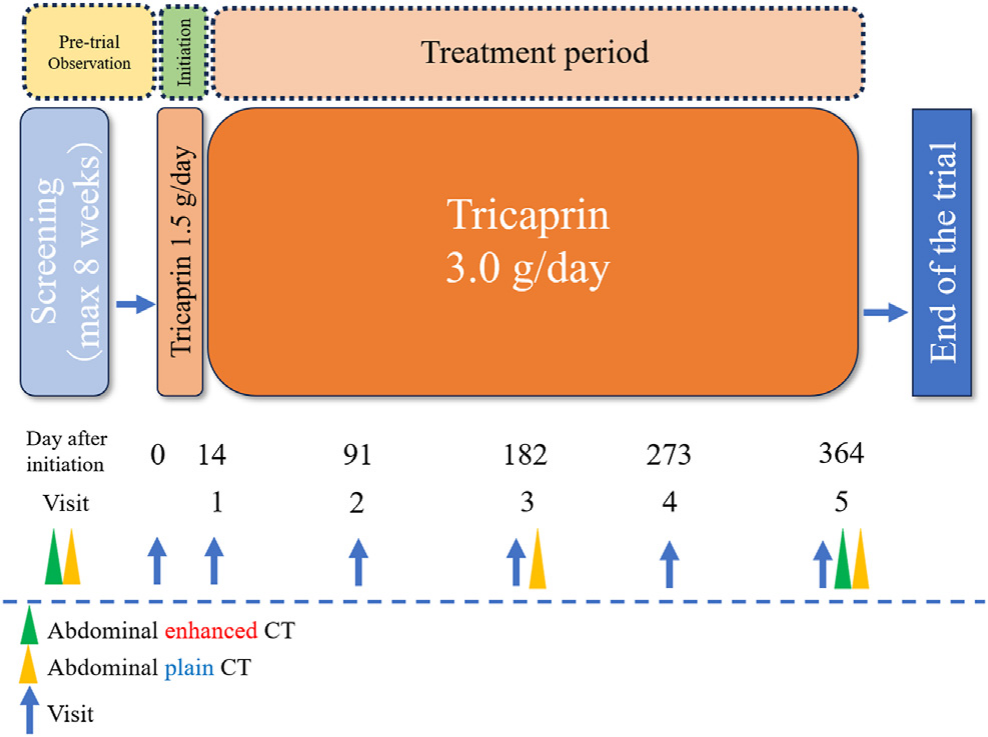
Trial scheme. The "pre-trial observation period" for screening before tricaprin administration shall not exceed 8 weeks. After tricaprin initiation, a 2-week follow-up period (initiation period) will follow, to rule out unexpected side effects. After initiation, patients will be monitored every 3 months and treated with tricaprin for 52 weeks. Contrast-enhanced computed tomography (CT) will be performed at the screening visit and visit 5, whereas unenhanced CT will be performed at the screening visit, visit 3, and visit 5.

This is a single-centre, open-label, and single-arm trial designed to investigate the safety of oral tricaprin 3 g/d for patients with AAA and explore potential indicators of efficacy. Patients with AAA who meet all the inclusion criteria and no exclusion criteria will be eligible for enrollment. A schematic representation of the trial is shown in Figure 1. Candidates will be recruited from an outpatient population diagnosed with asymptomatic infrarenal AAA. Tricaprin safety will be evaluated for all adverse events throughout the trial period. Additionally, efficacy indicators will be explored using abdominal CT findings. No post-treatment follow-up evaluation is planned.

### Study population

This study will include individuals aged 50-85 years who have a diagnosed small AAA under conservative follow-up treatment and will provide written informed consent. Eligible participants must have evidence of having a AAA with a maximum minor short-axis diameter (MMSD) of ≤ 45 mm on a contrast-enhanced CT scan performed within 5 weeks before or 8 weeks after they provide consent. All eligibility assessments will follow participant provision of informed consent. A total of 10 individuals will be included. Exclusion criteria include having any of the following: a prior aortic repair (endovascular or open); surgery for AAA scheduled for within a year; dissecting or severely calcified AAA; or an estimated glomerular filtration rate of < 30 mL/min. A complete list of eligibility criteria is provided in Table 1.

**Table 1:** Eligibility criteria

Inclusion criteria
• Aged 50–85 years at the time of consent
• Patients diagnosed with infrarenal AAA with a diameter of 30–45 mm according to enhanced CT scanning∗
• Provision of written informed consent directly from the patients
**Exclusion criteria**
• History of AAA surgical repair, including endovascular treatment
• Patients scheduled for surgical treatment for AAA within a year
• Symptomatic AAA
• AAA location excluding the infrarenal abdominal aorta
• Sac-type AAA
• Infected AAA or evident active aortitis
• Dissecting AAA
• Severely calcified AAA
• Heart failure symptoms of New York Heart Association classification III–IV
• Prognosis of < 2 y
• Estimated glomerular filtration rate ≤ 30 mL/min
• Bronchial asthma on treatment† or diagnosed with NSAIDs-exacerbated respiratory disorder
• History of contrast agent allergy
• AAA associated with connective tissue diseases (Ehlers–Danlos syndrome, Marfan syndrome, Loeys–Dietz syndrome)
• Consumed foods, including food oils labeled as “MCT” in their commercial names and containing medium-chain fatty acids, at least once daily for > 2 mo, and are unable to discontinue their use by the day before tricaprin initiation
• Diagnosed with familial hypercholesterolemia
• Unable to express their own will and manage the investigational substances on their own (eg, dementia)
• Others deemed ineligible for participation by the principal investigator or co-investigator

AAA, abdominal aortic aneurysm; CT, computed tomography; MCT, medium-chain triglyceride; NSAID, nonsteroidal anti-inflammatory drug.

∗ The CT Images performed up to 5 wk before or up to 8 wk after consent can be used for screening.

† Except for the cases without an asthma attack for > 5 y.

### Treatment protocol and study assessments

**Figure 2:**
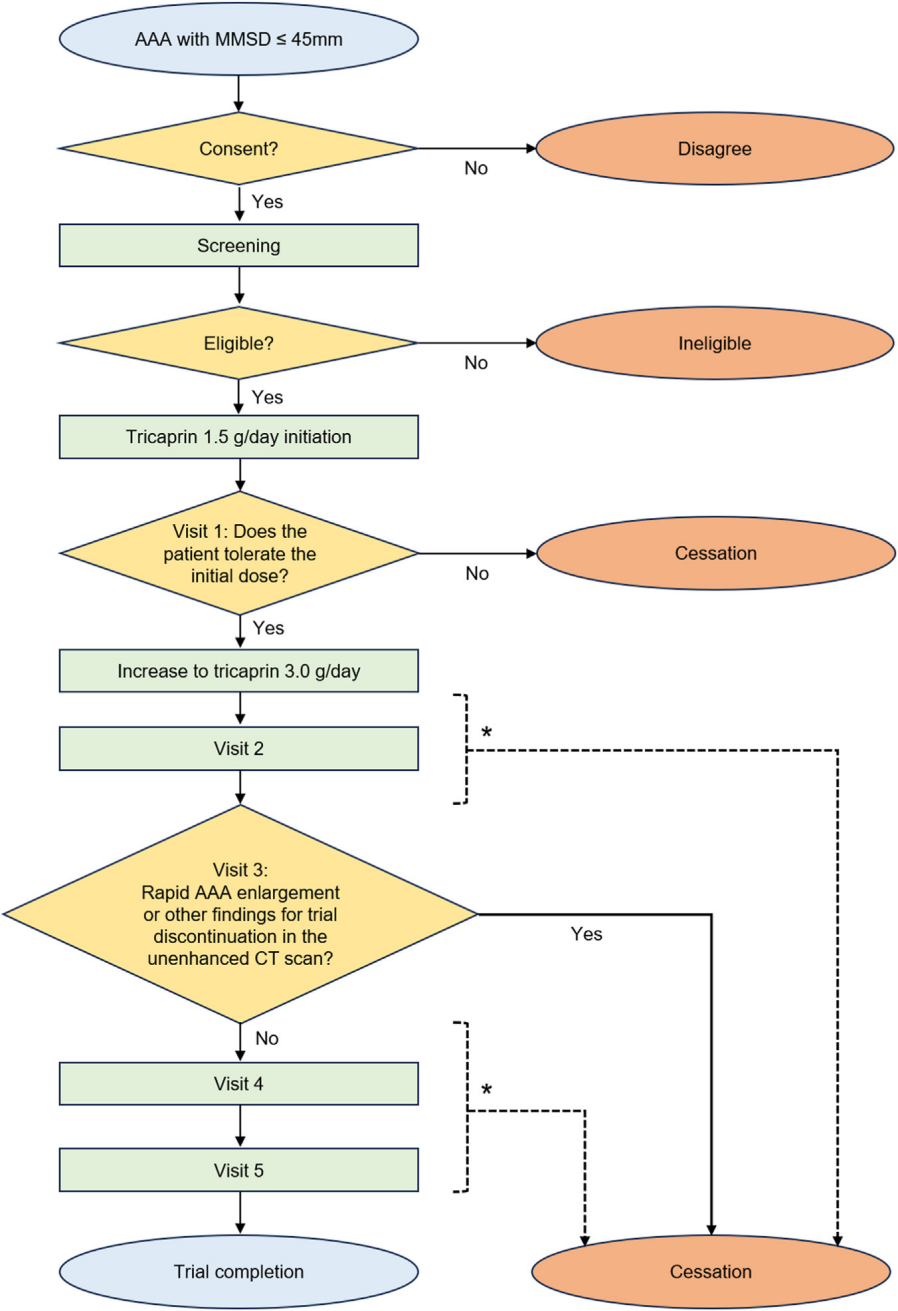
Patient flowchart. The patient flowchart outlines the trial process. All participants who provide informed consent will undergo screening tests. Patients will be excluded if they are found to be ineligible based on screening, unable to tolerate the increase in tricaprin from 1.5 to 3.0 g/d, show concerning computed tomography (CT) findings at visit 3, experience severe adverse events, request withdrawal, or are deemed ineligible at any visit for other reasons. AAA, abdominal aortic aneurysm; MMSD, maximum-minor short-axis diameter After initiating tricaprin use at 3, ∗ After initiating tricaprin use at 3.0 g/d, the trial may be discontinued if any abnormal findings warrant cessation, even between scheduled visits.

All participants will receive 1.5 g/d tricaprin for the first 2 weeks until visit 1, after which the dose will be increased to 3.0 g/d and maintained until week 52, administered in 3 divided doses per day. Participants unable to tolerate this increase will be excluded from further analyses. Lifestyle management, smoking cessation, and adjustment of metabolic and cardiovascular medications will be encouraged. Participants will visit the investigator quarterly to assess their symptoms, their experience of MACE, aneurysmal exacerbations, and side effects based on vital signs, adverse events, medication compliance, concomitant medication use, safety tests, and abdominal CT scans. Visits from visit 2 onward will be scheduled approximately every 3 months, with a 14-day flexibility period allowed. Contrast-enhanced and unenhanced CT scans at screening and visit 5 will measure the aneurysmal diameter, volume, Agatston score, and AAWCV. Visit 3 will include an unenhanced CT scan to check for abrupt expansion (≥ 5 mm over 6 months). Electrocardiograms will be conducted at the beginning and end of the trial to screen for asymptomatic cardiovascular complications. Tricaprin consumption will be monitored at each visit by using a patient diary and measuring the change in the weight of the tricaprin bottle. A tricaprin consumption rate of > 60% will be set as the threshold for the secondary endpoint sensitivity analysis. Figure 2 outlines the trial process (patient flowchart). Table 2 presents the data collection schedule.

**Table 2:** Data collection schedule

Study item, data collection, or activity	Informed consent	Screening visit	Day 0	Day 1	Visit 1	Visit 2	Visit 3	Visit 4	Visit 5	Cessation
Day of performance, d	—	–56 to –1	0	1	14	91	182	273	364	—
Allowance period, d	—	—	—	—	± 7	± 14	± 14	± 14	± 14	+ 28
Informed consent	X	X	—	—	—	—	—	—	—	—
Patient’s background	—	X	—	—	—	—	—	—	—	—
Eligibility confirmation	—	X	—	—	—	—	—	—	—	—
Tricaprin administration, g	—	—	—	1.5	➡	3.0	➡	➡	➡	—
Adherence check	—	—	—	—	X	X	X	X	X	X
Comorbidities/Medical history	—	X	—	—	—	—	—	—	—	—
Abdominal contrast CT scan	—	X	—	—	—	—	—	—	—	X
Abdominal unenhanced CT scan	—	X	—	—	—	—	X	—	—	X
Clinical findings		X	X	—	X	X	X	X	X	X
Laboratory test		X	X	—	X	X	X	X	X	X
Electrocardiogram		X	—	—	—	—	—	—	X	X
Adverse events		—	—							

The shorter arrows indicate the same dosages as those shown on the left side of each one. The longer arrow represents the continuous monitoring of adverse events between visits.

CT, computed tomography.

### Study endpoints and statistical assessments

The primary endpoint is the safety of oral tricaprin for 52 weeks, as determined by analysis of the occurrence rate of adverse events, specifically the following: (i) MACE incidence; MACE includes cardiovascular death, nonfatal myocardial infarction, and nonfatal stroke.; (ii) AAA exacerbation; adverse events related to AAA exacerbation include nonfatal acute abdomen with proven AAA rupture, nonfatal acute abdomen without overt AAA rupture, a sudden change in skin color, or edema of the lower limbs with severe pain, and an abrupt need for surgical intervention; and (iii) unpredictable tricaprin side effects; unpredictable tricaprin side effects include miscellaneous events presumably related to tricaprin use and not attributable to (i) or (ii). These events will be assessed via physical examinations, vital sign assessments, safety laboratory tests, physiological tests, and abdominal CT scans. In particular, for MACE, AAA exacerbation, and unpredictable side effects following administration of tricaprin, the number of occurrences and events and their percentages will be calculated and presented, apart from the total analysis of all adverse events, owing to their clinical importance. Additionally, vital sign measures and laboratory test results will be summarized with descriptive statistics at each visit as a subanalysis. Vital signs include the following: systolic blood pressure (mm Hg); mean blood pressure (mm Hg, calculated using the formula [diastolic pressure + (systolic – diastolic) / 3”]; and heart rate (beats per minute). Laboratory test results will include measures of levels of alanine aminotransferase, gamma-glutamyl transpeptidase, blood urea nitrogen, creatinine, sodium, potassium, chloride, D-dimer, C-reactive protein, and brain natriuretic peptide; a complete blood cell count (white, red, hemoglobin, hematocrit, and platelets); and estimated glomerular filtration rate.

We will evaluate AAA diameter, volume, Agatston score, and AAWCV as secondary endpoints. Data will be summarized using descriptive statistics and compared at each time point. For MMSD and AV, changes during the first 6 months post–tricaprin administration and the final 6 months prior to study completion will be calculated and summarized using descriptive statistics. The Agatston score will be analyzed by stratifying AV changes into 3 groups, as follows: progression, no change, and regression. No interim analysis will be conducted, and missing data will not be imputed. Primary and secondary endpoints are listed in Table 3.

**Table 3:** Primary and secondary outcomes

Primary (safety) outcome
• All adverse events
Additionally, the following items are to be collected, because of their clinical significance:
(i) MACE (cardiovascular death, nonfatal cardiac infarction, and nonfatal cerebral stroke);
(ii) adverse events related to AAA exacerbation (nonfatal acute abdomen with proven AAA rupture, nonfatal acute abdomen without overt AAA rupture, sudden skin color change or edema of lower limbs with severe pain, and abrupt need for surgical interventions); and
(iii) unpredictable side effects presumably related to tricaprin use (miscellaneous events that cannot be attributed to (i) and (ii).
**Secondary (exploratory) outcomes**
• AAA diameter (mm) and volume (cm^3^)
• Agatston score of AAA
• Aortic aneurysmal wall CT values (mm^3^, %)

AAA, abdominal aortic aneurysm; CT, computed tomography; MACE, major adverse cardiovascular events.

### Performance and measurements of CT scanning

Abdominal CT will be performed at 3 time points: the screening visit, visit 3, and visit 5. Contrast-enhanced CT after unenhanced CT will be performed at the screening visit and visit 5, whereas only unenhanced CT will be performed at visit 3. Imaging will be performed using a photon-counting CT device (NAEOTOM Alpha, Siemens Healthcare, Forchheim, Germany) following the major vessel CT protocol. For participants with an estimated glomerular filtration rate of ≤ 30 mL/min at visit 4, preventive measures with saline infusion for contrast-induced nephropathy will be considered at visit 5. If necessary, the contrast-enhanced CT scan at visit 5 will be replaced with an unenhanced CT scan to analyze MMSD and AV. All imaging analyses will be conducted using commercially available SYNAPSE VINCENT software (Fujifilm Medical Co., Tokyo, Japan) to ensure objective and reproducible measurements, with all analyses performed by a single observer; no additional observers will be assigned due to the proprietary nature of the algorithms, which have not been published by the developer to maintain objectivity. The analyses require 1 mm–thick sections of the entire AAA image.

The MMSD will be semi-automatically measured (in mm), employing straight multiplanar reconstruction images of the abdominal aorta at the level with the largest cross-sectional area of the aneurysm. These images will be oriented perpendicular to the aortic axis and obtained using the double-oblique^32^ and “outer-to-outer”^33^ wall methods. The area containing the MMSD will be suggested using a virtual stent deployment mode. However, if the observer identifies a larger MMSD in another adjacent area, the larger value will be adopted.

AV is the region from the lower edge of the left renal artery to the proximal bifurcation edge of the common iliac arteries. This value (in mm^3^) will be evaluated by summing the volumes of each of the 6 CT value fractions (mentioned below) visualized with a color map mode in the aortic multiplanar reconstruction images.

To assess AAA calcification, the Agatston score^34^ will be calculated automatically using the calcification analysis mode. The raw Agatston score obtained from the thin-section images will be adjusted by multiplying the score by 0.3333, as the original method of Agatston score used images with a 3-mm slice thickness.

Additionally, AAWCV—the total volume of regions with specific CT values and their proportions to the AV—will be evaluated in mm^3^, and percentages, respectively. The 6 ranges mostly encompass all aneurysm components: (i) -100 ≤ Hounsfeld units (HU) < -40; (ii) -39 ≤ HU < -10; (iii) -9 ≤ HU < 20; (iv) 21 ≤ HU < 50; (v) 51 ≤ HU < 80; and (vi) 81 ≤ HU. AAWCV will be evaluated within each range above and in the early and delayed phases of contrast-enhanced CT imaging.

### Criteria for cessation of the protocol treatment

Tricaprin administration will be discontinued, and urgent surgical intervention will be considered if any of the following occur: aneurysmal diameter increases by ≥ 5 mm over a 6-month period or MMSD exceeds 51 mm on CT scans asymptomatically; AAA-related abdominal symptoms develop; or imaging evidence suggests impending rupture, even without symptoms. The need for urgent surgical intervention will be assessed in all patients who meet these criteria. However, participants meeting these criteria may opt to continue tricaprin use until the day of surgery.

### Determination of the target for data analysis

The full analysis set will be used for data analysis, including all patients, excluding ineligible cases, with at least one tricaprin administration of 3.0 g/d and visit, regardless of treatment discontinuation during the follow-up period. A sensitivity analysis of secondary endpoints will be scheduled for participants with good compliance to medication use, defined as a tricaprin consumption rate > 60%. This threshold is based on phase 2a trial data,^29^ which showed that patients with triglyceride deposit cardiomyovasculopathy who maintained a tricaprin intake of ≥ 1.5 g/d showed improved clinical outcomes. If participants consume > 60% of tricaprin throughout the trial period, they will have ingested an average daily dose exceeding 1.8 g/d. Therefore, individuals in the sensitivity-analysis group should be considered to have received a dose sufficient to assess tricaprin efficacy.

### Sample size calculation and statistical considerations

A sample size of 10 participants was selected based on ethical considerations and the exploratory nature of the study. Although medium-chain triglycerides have a well-established safety profile, the safety of tricaprin in patients with AAA remains unverified. Moreover, including many participants in an initial safety assessment trial is unethical. AAAs are clinically diverse, with varying progression rates, and no established efficacy endpoints. Although MMSD is used frequently, sometimes it may not increase over extended periods.^35^ This variability makes the establishment of its clinical significance challenging. Given these factors and the lack of spontaneous reverse remodelliing in AAAs, we have opted not to perform statistical significance tests. Instead, we will summarize our findings using descriptive statistics.

### Time frame

Patient recruitment began on May 17, 2024, and it is expected to be completed by November 30, 2024. The study is anticipated to be completed by March 31, 2026.

### Study organization

The trial was designed by the Laboratory of Cardiovascular Disease, Novel, Non-invasive, and Nutritional Therapeutics (CNT), Department of Triglyceride Science, Graduate School of Medicine, Osaka University, Osaka, Japan. The Department of Cardiovascular Surgery and Cardiovascular Medicine at Osaka University Hospital will propose a backup plan for patient enrollment and the management of emergency cases. The Department of Radiology at Osaka University Hospital, will analyze the CT images. Data management, statistical analysis, and monitoring will be performed at the Department of Medical Innovation, Osaka University Hospital, Osaka, Japan, as part of the second Clinical Research Support Project of Osaka University Hospital. The Tochino Foundation will provide all investigational substances at no cost. The authors are responsible for the trial conception, manuscript preparation and editing, and final content.

### External committee

The trial will be conducted in compliance with the ethical principles of the Declaration of Helsinki and the Clinical Research Act. Approval was obtained from the Osaka University Clinical Research Review Committee on May 9, 2024, with the approval number of CRB5180007. Written informed consent will be obtained from all participants. The trial registration number is jRCTs051240036 from the Japan Registry of Clinical Trials. No patients with AAA were engaged in developing the study protocol or steering the committee.

## Discussion

### Study rationale, implication, and anticipated results

This study aims to provide new insights into the pathophysiology of AAA and treatment gaps using a unique evaluation method. We expect to establish the safety of tricaprin use for treating patients with AAA, and we propose a clinically feasible pathologic evaluation method using the AAWCV and the Agatston score.

In the aortic hypoperfusion model, tricaprin administration significantly reduced aneurysm diameter to near-normal levels and demonstrated high vasa vasorum patency with minimal proliferation. We also observed decreased hypoxia-inducible factor 1α levels and improved vascular smooth muscle cell function and abundance.^23^ These findings suggest that tricaprin use can maintain aortic wall blood flow via the vasa vasorum, enhance medial smooth muscle cell function, and reduce lipid accumulation. To validate the effects of tricaprin on AAA, histologic confirmation of the improved blood flow and lipid reduction in human specimens is necessary. Because of the challenges in obtaining human AAA specimens from patients undergoing follow-up care, establishing noninvasive evaluation methods is essential. We aim to verify the potential of the AAWCV for evaluating aortic wall contrast as a substitute for pathologic analysis, as lipid reduction and blood flow improvement are expected to alter the contrast CT values within the aortic wall.

### Potential challenges, mitigation strategies, and comparison with previous studies

Many RCTs have faced challenges in interpreting results owing to nonstandardized AAA measurement methods and a lack of established criteria for evaluating investigational drug efficacy.^9^, ^10^, ^11^, ^12^, ^13^, ^14^, ^15^, ^16^ Establishing reproducible methods for measuring AAA^36^ and appropriate techniques for indirectly assessing histologic treatment effects is crucial. We will use high-resolution photon-counting CT images and semiautomated analysis of the AAA short diameter and volume using 1 mm–thick slices to minimize measurement errors. Additionally, we will perform AAWCV analysis as a noninvasive measure that reflects the histologic state of AAA to determine treatment efficacy. This highly objective and easily implementable analysis method with minimal examiner subjectivity is a potential candidate for efficacy evaluation criteria.

### AAWCV, a novel evaluation method for CT findings on AAA

AAWCV evaluation is crucial for 2 reasons. First, the adipocyte count in the aneurysm adventitia correlates with expansion and rupture rates, linking quantitative CT evaluation of lipid accumulation to the AAA state.^31^^,^^37^ Second, aortic hypoperfusion, a key factor in AAA development, may affect aneurysm wall contrast enhancement. Studies have suggested a correlation between aortic wall enhancement and post–endovascular surgery reverse remodelliing,^38^^,^^39^ supporting our hypothesis. High contrast enhancement may indicate good AAA wall blood flow; favourable changes in reverse remodelling and AAWCV may be observed following tricaprin administration. Observed reverse remodelling cases would require comprehensive AAWCV analysis and group comparisons to identify the influencing factors. If ectopic adipocytes or impaired blood flow areas are identified and quantified on enhanced CT, histologic improvement in AAA after tricaprin treatment may be inferred from the changes in CT findings. Thus, AAWCV may be a partial alternative for a histologic assessment. However, even with the high spatial resolution of photon-counting CT, selectively quantifying these areas remains difficult. Additionally, chronic inflammation, a key factor in AAA pathogenesis, may appear as delayed contrast^40^ on enhanced CT, complicating the prediction of whether the change in contrast values will increase or decrease. Therefore, we plan to classify all CT values within AAA and evaluate the changes in volume in each range to assess their potential as a surrogate marker for AAA pathologic analysis.

### Agatston score as an indicator of histologic reversibility

For exploratory purposes, we plan to evaluate AAA calcification. Previous studies have suggested a relationship between aortic aneurysms and calcification scores, showing associations with cardiac event risk^41^ and accelerated AAA expansion risk.^42^ However, debate remains regarding whether severe calcification worsens rupture rates or expansion speeds, challenging the notion that it should be considered a candidate for efficacy validation on its own. We predict that as an aneurysm becomes more calcified, its plasticity decreases, making regression detection less likely and resulting in minimal improvement in the lower CT value fraction of the AAWCV. Therefore, we intend to compare the Agatston scores before and after the intervention to determine whether these scores reflect the histologic alterations and reversibility of AAA. Evaluating trends in quantitative vascular calcification scores across the 3 groups, particularly in the “regression” group, is important as a predefined endpoint. Additionally, concurrently analyzing the favourable changes in AAWCV (with a reduction in low CT value regions) and decreases in the Agatston score may provide insights into the relationships among changes in adipocyte-rich, ischemic, and calcified regions within the aortic wall. Therefore, assessing the Agatston score across these 3 groups could guide the indirect histologic evaluation of AAA.

### Study limitations

This study has 4 major limitations. First, we will use a small sample size and a short evaluation period to ensure the safety of the participants. A control group will not be included because of the lack of effective AAA treatments and the rarity of spontaneous regression.^43^ Thus, discussions on direct effectiveness based on trial results are complicated. Caution should be taken when discussing efficacy, particularly if AAA reverse remodelliing is observed, aside from its significant clinical impact. Moreover, to minimize the risk of withdrawal, at the eligibility assessment phase, we will estimate aneurysm diameter progression during this trial based on AAA growth rates from available data over the past 2-3 years. We also will assess high-risk findings for aortic dissection, such as ulcer-like projections, and confirm primary or secondary prevention of ischemic heart disease before enrollment. We will use these factors to guide the selection for reducing dropout rates. Furthermore, although aiming to identify efficacy endpoints with a small sample size of 10 patients may seem ambitious, prior studies lack established methods for evaluating efficacy, as no cases of aortic aneurysm regression have been observed with interventions. Additionally, the absence of a standard of care prevents determination of a clinical efficacy rate, complicating the calculation of the sample size required to demonstrate statistical significance. Given the rarity of annual hard event rates,^44^ efficacy evaluations cannot proceed without aneurysmal regression being achieved first through an intervention. Therefore, this study has not been designed to demonstrate efficacy but rather to serve as a foundation for larger clinical trials. In addition, a post-treatment follow-up protocol has not been established because the short half-life of tricaprin in humans (< 2 h) makes long-term adverse events from its accumulation unlikely. Indeed, no adverse events have been reported in AAA animal models treated with tricaprin, and the product has already been marketed in Japan without serious safety concerns. Therefore, extending the follow-up period beyond the clinical study period is unlikely to yield additional valuable information regarding tricaprin safety.

Second, although young male rats were used in the aortic hypoperfusion model, most participants are expected to be aged > 70 years. Age-related decline in tissue regeneration and the estimated AAA duration should be considered when evaluating the results, particularly those that are negative.

Third, the inclusion criteria allow for significant variability among participants, which could result in strong heterogeneity in the baseline risk of adverse events. However, we have demonstrated already that tricaprin can be used safely in patients with triglyceride deposit cardiomyovasculopathy and concomitant metabolic conditions, such as diabetes mellitus and hypercholesterolemia. This finding has been demonstrated further in previous clinical trials with heterogeneous populations.^29^ Furthermore, tricaprin capsules have been commercially available in Japan since 2020 and have been taken by a wide range of consumers across various age groups without reports of serious side effects. Accordingly, we purport that the heterogenous age range and AAA diameter will not impact the assessment of potential adverse effects of tricaprin use significantly.

Fourth, other unknown factors not incorporated into the exclusion criteria may affect AAA. For example, we will not evaluate metformin^45^ and statin^46^ use, which reportedly correlate with a 0.8-mm per year reduced AAA worsening rate. However, this level falls within the range of imaging modality variability and measurement error, making the difference statistically significant but not clinically meaningful. Meanwhile, no clear evidence has been found that the use of other drugs affects the progression of aortic aneurysms. Excluding use of particular drugs could potentially worsen the management of key conditions, such as ischemic cardiac disease, which frequently coexist with AAA. Therefore, the use of specific medications will not be excluded. Similarly, specific nutritional components will not be excluded, as none have been shown to have greater efficacy than tricaprin in animal studies with AAA.

### Conclusions

To our knowledge, F-HAAAT is the first clinical study to examine the safety of tricaprin use in human AAA and to explore the indicators of its efficacy. The results of this study may expand noninvasive treatment options for patients with AAA and provide new insights into clinicopathologic evaluation methods.
